# From pathogens to policy: using network analysis to map the knowledge base on human–zoonotic disease dynamics underpinning global pandemic policy

**DOI:** 10.1186/s12961-025-01434-5

**Published:** 2025-11-29

**Authors:** Bruna de Paula Fonseca, David Bell, Garrett Wallace Brown

**Affiliations:** 1 Center for Technological Development in Health (CDTS), Oswaldo Cruz Foundation (Fiocruz), Rio de Janeiro, RJ Brazil; 2 Independent Global Public Health Consultant, Lake Jackson, TX United States of America; 3 School of Politics and International Studies (POLIS), University of Leeds, Leeds, United Kingdom

## Abstract

Zoonotic disease dynamics (ZDD), encompassing pathogen spillover, transmission pathways and host–pathogen interactions, are widely acknowledged as drivers of emerging infectious diseases. Yet, the extent to which recent pandemic prevention, preparedness and response (PPPR) policies – the integrated frameworks guiding international efforts to anticipate and manage infectious disease threats – are grounded in this expanding body of scientific research remains unclear. This study examines how research on ZDD is cited in six influential global policy reports published between 2021 and 2023. We employed citation network analysis and qualitative profiling to compare references cited in these reports with those cited by a systematically identified set of broad-scope scientific publications on ZDD. Of the 313 references across the six reports, only 59 (19%) pertained to ZDD, a relatively small proportion considering that zoonotic diseases are framed as primary drivers of pandemic risk in these same reports. The academic literature is highly diverse and predominantly focused on specific pathogens (66%), with few studies offering a broad-scope perspective (4%) that addresses the complexity of ZDD. The citation network of the selected broad-scope literature was fragmented revealing low convergence of the knowledge base. Shared references between reports and scientific literature (*n* = 31) were mostly modelling studies (45%) or reviews (35%). Secondary data predominated (45%), and only 6% relied primarily on original field or laboratory data. Foundational studies were often overrepresented. This narrow and selective evidence base risks obscuring key uncertainties and limiting the diversity of perspectives that inform global PPPR strategies. Our findings highlight the value of more systematic approaches to scientific evidence use in PPPR policy documents. Strengthening the science–policy interface in PPPR requires greater engagement with emerging research, epistemic diversity, and the acknowledgment of uncertainty – essential steps toward building more adaptive, equitable and resilient strategies.

**Supplementary Information:**

The online version contains supplementary material available at 10.1186/s12961-025-01434-5.

## Background

In recent years, pandemic prevention, preparedness and response (PPPR) – the integrated approach encompassing surveillance systems, risk mitigation strategies and outbreak response mechanisms – has become a central theme in global health policy. The coronavirus disease 2019 (COVID-19) pandemic increased the prominence of coordinated international strategies to prevent, detect and respond to infectious disease threats, and catalysed a growing body of research on pandemic risk reduction [1, 2]. Among the areas gaining prominence, understanding the processes of zoonotic pathogen spillover, transmission and emergence has become a critical focus for risk mitigation. In this study, we use the term “zoonotic disease dynamics” (ZDD) to refer to the multidisciplinary research examining these processes, including: spillover mechanisms, transmission pathways, host–pathogen interactions, infection dynamics at the human–animal interface, and epidemiological patterns of zoonotic disease emergence and spread [3, 4]. Major global organizations, including the World Bank (WB), the WHO and the Group of Twenty (G20), have emphasized the importance of addressing zoonotic risks as part of broader pandemic prevention efforts, often highlighting the relevance of One Health and integrated surveillance approaches [5–7].

While the scientific literature on ZDD has expanded considerably, less is known about how this body of knowledge is reflected in major policy frameworks. The integration of scientific evidence into policy is critical not only for ensuring that interventions are well-founded but also for fostering transparency, accountability and legitimacy in global health governance [8]. Yet, the pathways through which scientific studies influence high-level policy outputs, whether through citations, conceptual framing or selective use of evidence, remain underexamined. Recent critiques have raised concerns that the evidence base for PPPR strategies may be uneven, overly reliant on a narrow set of studies and potentially limited in its empirical grounding [9, 10].

Bibliometric and citation network analysis methods offer valuable tools for examining how evidence circulates within and across scientific and policy domains [11, 12]. These methods allow researchers to explore policy evolution [13], map evidence sources and identify influential publications in policy documents [14], as well as trace the policy–science interface [15, 16]. This is particularly relevant in emerging interdisciplinary fields such as ZDD, where consensus is still developing and knowledge production is distributed across diverse disciplines [17].

To explore the science–policy interface in this domain, this study examines how scientific literature on ZDD is cited and mobilized in influential global PPPR reports that have shaped recent global health agendas. By analysing the references cited in these reports and comparing them with a systematically identified subset of broad-scope scientific publications on ZDD, we use citation network analysis and qualitative profiling to examine the knowledge base informing current policy discourse.

Our analysis contributes to ongoing efforts to better understand the science–policy interface in global health, particularly in complex and evolving fields. By examining which studies are cited in key global reports, how often they are referenced and what kinds of contributions they make, we offer a structured perspective on the alignment and potential disconnects between scientific literature and the evidence base informing PPPR.

Our findings reveal that recent PPPR reports engage only partially with the available scientific literature on ZDD, with some reports having no citations, while others tended to cluster around key sources. Most of the scientific literature remains focused on specific pathogens, with relatively few studies addressing the systemic complexity of ZDD. This integrative literature is also conceptually fragmented, with little convergence around shared frameworks or findings. The subset of references cited by both reports and scientific publications tends to privilege modelling studies and reviews on the basis of secondary data, while empirical, field-based research is largely absent. Foundational studies appear frequently across sources, raising concerns about citation concentration and the selective nature of evidence use. These patterns underscore the need for more transparent, inclusive and critically informed strategies for using scientific knowledge in global health policy-making.

## Methods

### Selection of global reports

Our study was guided by the following research question: How do major global PPPR policy reports cite scientific literature on zoonotic disease processes, and what do these citation patterns reveal about the breadth, diversity and empirical grounding of the knowledge base informing these frameworks?

To examine this question, six influential reports on PPPR were selected on the basis of criteria established in a previous analysis [9]. Three reports were produced to guide pandemic preparedness deliberations at The Group of Twenty (G20) meeting in 2022 and formed a basis for costing of the WHO pandemic agenda, as well as one World Bank 2022 report further highlighting additional estimates for One Health interventions not covered in these, and two subsequent 2023 WHO publications outlining revised (post-COVID) WHO recommendations on pandemic and epidemic preparedness and response. Together, these six reports form the basis for these organizations’ approaches to addressing pandemic risk:

- World Bank Report (2022): *Putting pandemics behind us* (WB Pandemics) [5]
- World Bank Report (2022): *Increasing investments in One Health to reduce risks of emerging infectious diseases at the source* (WB One Health) [6]
- Report of the G20 High Level Independent Panel (HLIP) on financing the global commons for pandemic preparedness and response (2021): *A global deal for our pandemic age* (G20 Global Deal) [18]
- WHO–World Bank Report (2022): *Analysis of pandemic preparedness and response architecture, financing needs, gaps and mechanisms* (WHO–WB PPR) [7]
- WHO Report (2023): *Future surveillance for epidemic and pandemic diseases: a 2023 perspective* (WHO Surveillance) [19]
- WHO Report (2023): *Managing epidemics: key facts about major deadly diseases* (WHO Managing Epidemics) [20]

### Screening of references in reports

All references cited in the reports were manually reviewed by a public health expert (B.F.) to assess relevance to ZDD with additional input and review (D.B.). Inclusion criteria targeted references addressing: (i) zoonotic transmission pathways; (ii) infection mechanisms or host–pathogen interactions; (iii) spillover events from animal reservoirs to humans; and/or (iv) epidemiological elements such as surveillance or outbreak modelling. Only references directly aligned with these themes were retained for further analysis.

### Mapping the scientific literature

Scientific literature on ZDD was retrieved from the Scopus database (Elsevier) using three targeted queries focusing on different dimensions of ZDD, especially those related to human health. Searches were limited to titles and abstracts for higher specificity. Publication dates were restricted to 2018–2022 to ensure relevance to selected global reports and capture research likely to have influenced the PPPR reports and policy recommendations.

Query 1: Cross-species and interspecies transmission

TITLE-ABS ((“cross-species spillover”) OR (cross-species PRE/1 infection) OR (cross-species PRE/1 transmission) OR (“cross-species spill over”) OR (“interspecies spillover”) OR (interspecies PRE/1 infection) OR (interspecies PRE/1 transmission) OR (“interspecies spill over”)) AND TITLE-ABS (human)

Query 2: Zoonotic spillover events

TITLE-ABS (zoonotic PRE/1 spillover) OR (zoonotic PRE/1 “spill over”) OR (“spillover of zoonotic pathogens”) OR (“spill over of zoonotic pathogens”) OR (“infectious disease spillover”) OR (“infectious disease spill over”) OR (“spillover event”) OR (“spill over event”) OR (“pathogen spill over”)

Query 3: Emergence and Re-emergence of Zoonotic Diseases

TITLE-ABS (“emerg* zoonotic disease”) OR (“zoonotic disease emergence”) OR (“emergence from zoonotic reservoirs”) OR (“zoonotic disease outbreak”) OR (“newly emergent zoonos*s”) OR (“re-emerg* zoonos*s”) OR (“newly emergent zoonotic disease”) OR (“re-emerg* zoonotic disease”) AND TITLE-ABS (human)

### Relevance screening of scientific literature

Titles and abstracts of retrieved scientific publications were manually categorized following a hierarchical classification system (Table 1). Each scientific publication was assessed sequentially from the first category (e.g. companion animals) downwards. Once assigned to a category, publications were not reclassified, even if potentially relevant to multiple categories, to maintain consistency. The full categorized dataset is available in Additional File 1.

**Table 1:** Thematic categorization of ZDD publications

Category	Definition
Companion animals	Role of pets (dogs, cats) in zoonotic disease transmission
Specific diseases	Studies on specific diseases/pathogens (e.g. rabies, coronaviruses, influenza, among others)
Epidemiology, surveillance, transmission	Research on endemicity, molecular epidemiology, genetic profiles, serosurveys, spatial epidemiology, mathematical modelling of transmission, prevalence
Genetics	Genome sequencing, genetic characteristics, molecular characterization, molecular detection, phylogenetic analysis, whole-genome sequencing, genetic diversity
Infection, diagnostics, vaccines	Studies on infection mechanisms, diagnostics or vaccine development
Broad-scope/relevant	Broad studies on zoonotic disease emergence or spillover, not restricted to specific diseases, with a focus on the intersection of zoonotic spillover with environmental, societal and global health factors, and emphasis on understanding and preventing future pandemics
Out of scope	Studies not fitting any of the above categories

### Citation network analysis

Citation networks were built using Gephi version 0.10.1 [21], with each node representing a document (either a scientific publication, a global policy report or a cited reference). Directed edges indicate citation links, pointing from the citing document to the cited one.

While the selection of scientific publications was limited to a defined time frame (2018–2022), the references cited within those publications, as well as those cited by policy reports, were included in the analysis regardless of their year of publication. This approach ensured that foundational or widely cited studies were captured in the analysis, even if published before the selected time frame, providing a more complete view of the knowledge base informing recent research and policy.

To assess the relative importance of individual scientific publications within the network, we applied in-degree centrality [22], measuring the number of times a publication was cited by others in the network. Publications with higher in-degree centrality are those that were more frequently cited within the dataset and can be interpreted as having greater influence or visibility in the scientific or policy discourse under analysis.

While this metric provides insight into citation prominence, it does not measure the interpretive weight or policy relevance assigned to a given study. The analysis captures structural patterns of citation, not the substantive influence of the cited evidence.

### Qualitative analysis of shared references

The references cited by both global policy reports and broad-scope research on ZDD were read in full and analysed across three analytic dimensions: type of contribution, type of data and thematic scope. Categorization was performed by B.P.F. on the basis of predefined interpretive criteria described as follows:

i. Type of contribution: (a) review/synthesis: narrative or systematic reviews that summarize existing knowledge; may propose frameworks, hypotheses or conceptual models without presenting new data; (b) empirical/field or lab-based: studies that generate new primary data through laboratory experiments, fieldwork or direct observation; (c) empirical/modelling or simulation: studies that use statistical/computational models or literature data to test hypothesis or simulate or predict different aspects of ZDD; (d) mixed: papers combining multiple types of contributions.
ii. Type of data: (a) primary data: generated directly by the authors through original research; (b) secondary data: reused from existing datasets, publications or databases; (c) no data: theoretical or argumentative papers without empirical analysis; (d) mixed: combination of any of the above types.
iii. Thematic scope: (a) broad: studies addressing general patterns, mechanisms or drivers of zoonotic emergence, often across multiple pathogens, regions or systems; (b) specific: studies focused on a particular pathogen, host species, outbreak event or geographic context.

In cases where a reference included multiple methodological components or ambiguous characteristics, “mixed” classifications were accommodated. A full list of shared references and their classification is presented in Additional File 2. Descriptive statistics were generated to support interpretation and visualized accordingly.

## Results

### Use of zoonotic disease dynamics (ZDD) evidence in global pandemic reports

The reference lists of six key global reports advocating on pandemic preparedness were analysed to assess the presence of scientific evidence on zoonotic disease dynamics (ZDD). Across the reports, 313 total references were cited, of which 70 were related to ZDD. After removing duplicates, 59 unique ZDD-related references remained (Table 2).

**Table 2:** References on ZDD cited in global reports on pandemic preparedness

Report	Total references	ZDD references
WB Pandemics (2022)	92	28
WB One Health (2022)	73	34
G20 Global Deal (2021)	27	0
WHO–WB PPR (2022)	10	2
WHO Surveillance (2023)	111	6
WHO Managing Epidemics (2023)	0	0
Total (all reports)	313	70
Unique ZDD references	–	59

Notably, one report – WHO Managing Epidemics – contained no references, and another – G20 Global Deal – did not cite any ZDD-related literature. Therefore, the analysis focused on the remaining four reports: WB Pandemics (2022), WB One Health (2022), G20 PPR (2022) and WHO Surveillance (2023).

### Citation patterns and shared references across reports

**Fig. 1:**
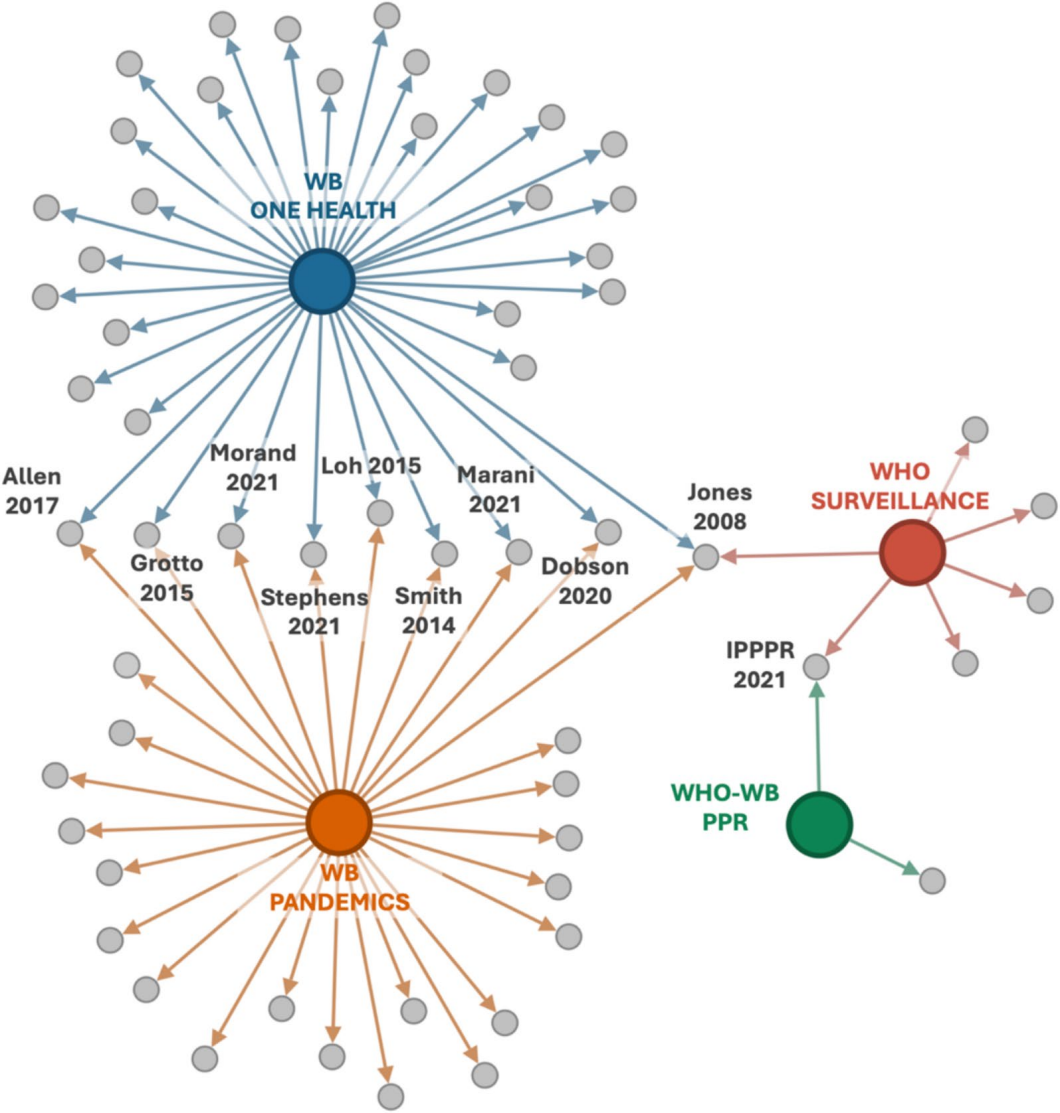
Citation network of ZDD references in four global reports. Coloured nodes of larger size represent reports, and grey nodes represent ZDD-related references. Directional links indicate citations. Blue: World Bank Report 2022 – *Increasing investments in One Health to reduce risks of emerging infectious diseases at the source* (WB One Health); orange: World Bank Report 2022 – *Putting pandemics behind us* (WB Pandemics); green: WHO–World Bank Report 2022 – *Analysis of pandemic preparedness and response architecture, financing needs, gaps and mechanisms* (WHO–WB PPR); red: WHO Report 2023 – *Future surveillance for epidemic and pandemic diseases: a 2023 perspective* (WHO Surveillance)

The citation network shown in Fig. 1 maps the relationships between the four analysed global reports and the ZDD-related references they cite. Each report is represented as a colour-coded node, connected by links to the references they cite.

The World Bank reports (WB Pandemics and WB One Health) cite the most ZDD-related studies. Several references are shared between these two, suggesting a common evidentiary base. In contrast, WHO–WB PPPR and WHO Surveillance reports show fewer citations and limited overlap with the World Bank reports, suggesting a more fragmented or selective engagement with ZDD evidence.

Some references appear in multiple reports, highlighting their status as foundational sources (e.g. Jones et al., [23]). These shared citations suggest emerging consensus around key studies perceived to be most relevant to ZDD and pandemic preparedness.

### Identifying broad-scope scientific literature on ZDD

**Fig. 2:**
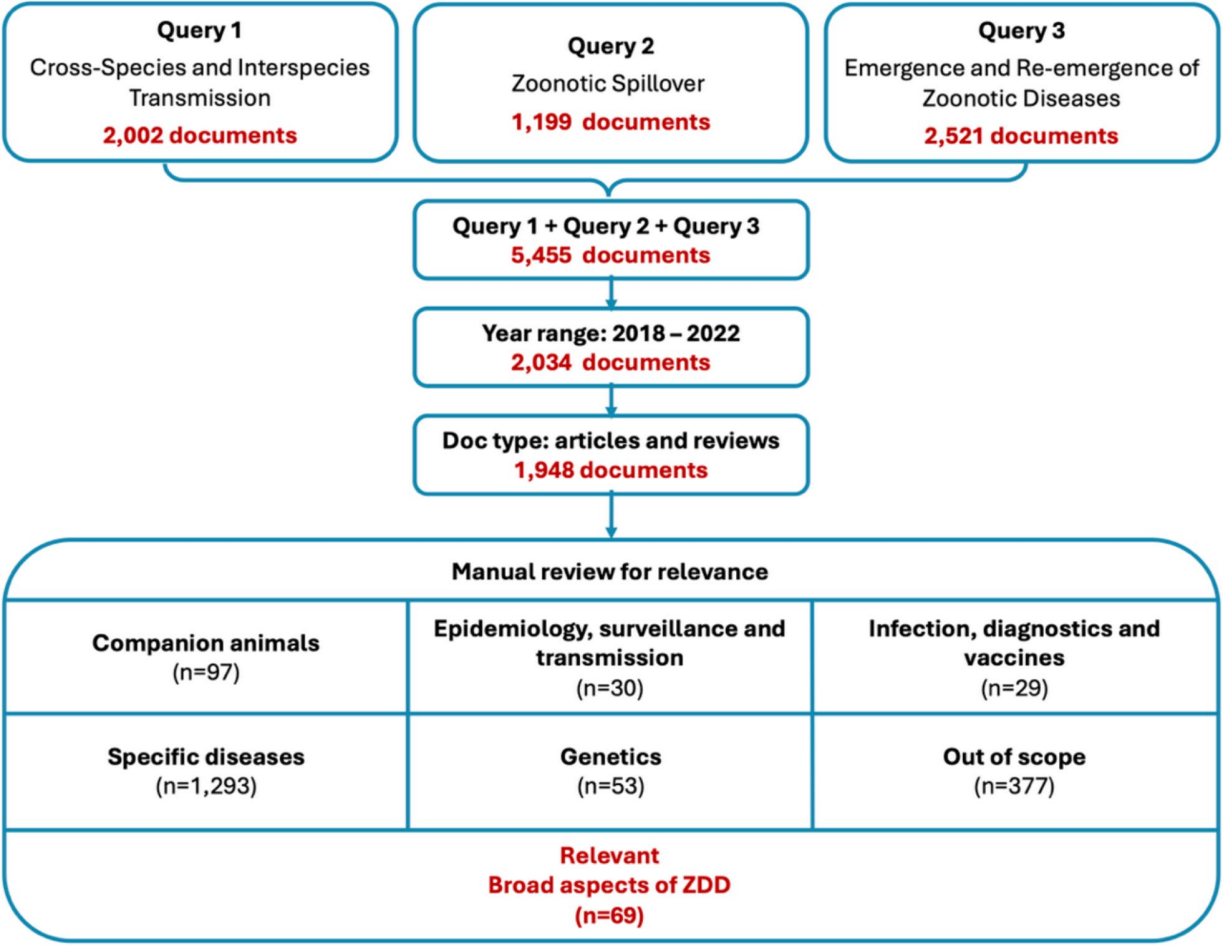
Flow diagram illustrating the stepwise selection process used to identify broad-scope scientific literature on ZDD

To identify scientific work most likely to have informed global policy-making, a structured search for broad-scope ZDD literature was conducted (Fig. 2). A combined query targeting cross-species transmission, zoonotic spillover and zoonotic disease emergence returned 5445 results. After filtering for articles and reviews published between 2018 and 2022, 1948 publications remained. Manual screening identified 69 broad-scope articles (4%), targeting comprehensive aspects of ZDD and offering integrative perspectives beyond specific pathogens (see dataset in Supplementary Material). The common thread is the intersection of zoonotic spillover with environmental, societal and global health factors, with an emphasis on understanding and preventing future pandemics.

### Foundational studies guiding the broad-scope ZDD discourse

**Fig. 3:**
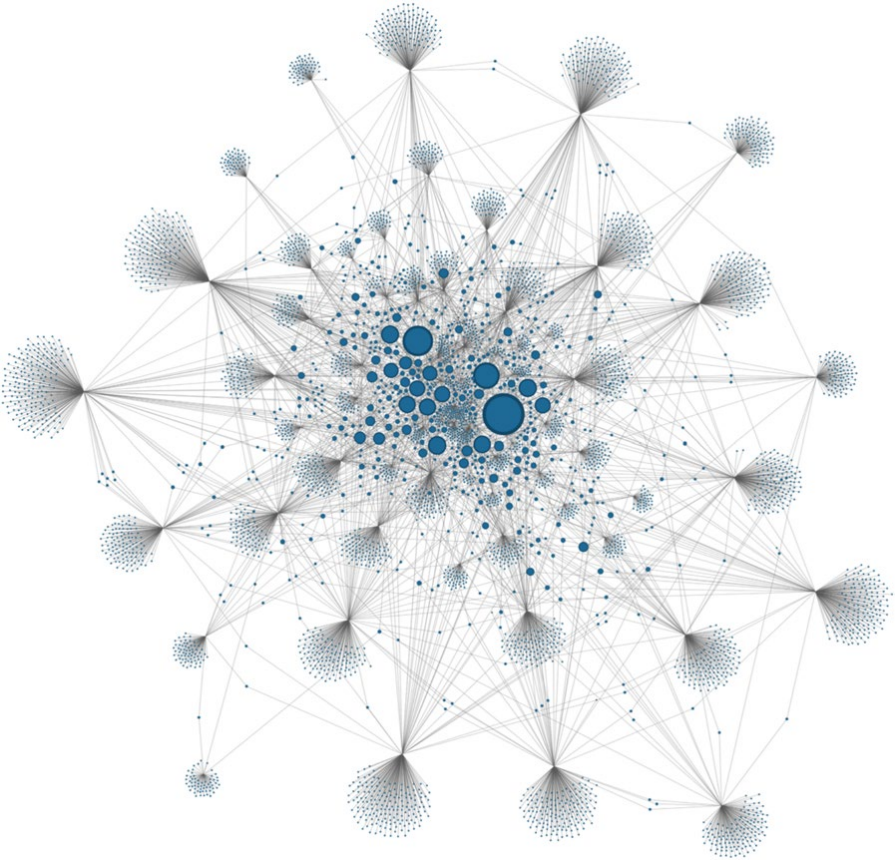
Citation network of the selected broad-scope ZDD literature subset (*n* = 69). Each node represents an individual article, and directed edges indicate citation relationships between them. Node size is proportional to the number of times an article was cited within this dataset (in-degree centrality), highlighting the most influential publications

The broad-scope literature subset was further analysed through citation network mapping. Figure 3 shows the network, including 5021 nodes – comprising the 69 focal papers and 4952 cited references – and 6302 edges indicating citation relationships.

The resulting network reveals a fragmented structure, characterized by multiple small, loosely connected clusters. This fragmentation reflects the high diversity of sources: 87% of all cited references were mentioned by only one paper, suggesting a field that is conceptually broad.

A few central studies – represented by larger nodes in the network – were cited across a portion of the dataset, functioning as so-called conceptual anchors for the broad-scope ZDD discourse (Table 3). The most cited reference is Jones et al. [23], cited by 58% of the papers, reflecting its foundational role in framing global patterns of emerging infectious diseases. This is followed by Plowright et al. [3] and Olival et al. [24], cited by 42% and 36% of the papers, respectively, both of which provide key conceptual and empirical contributions to understanding zoonotic spillover pathways and risk factors. Several other papers appear in 22–25% of the citing documents, including Jones et al. [25], Morse et al. [26], and Lloyd-Smith et al. [27], highlighting recurring attention to the environmental, ecological and epidemiological drivers of zoonotic emergence.

**Table 3:** Top five most cited references in broad-scope ZDD literature

Rank	Author	Title	Times cited (%)
1	Jones et al. [23]	*Global trends in emerging infectious diseases*	40 (58%)
2	Plowright et al. [3]	*Pathways to zoonotic spillover*	29 (42%)
3	Olival et al. [24]	*Host and viral traits predict zoonotic spillover from mammals*	25 (36%)
4	Jones et al. [25]	*Zoonosis emergence linked to agricultural intensification and environmental change*	17 (25%)
4	Morse et al. [26]	*Prediction and prevention of the next pandemic zoonosis*	17 (25%)
4	Lloyd-Smith et al. [27]	*Epidemic dynamics at the human animal interface*	17 (25%)
5	Keesing et al. [4]	*Impacts of biodiversity on the emergence and transmission of infectious diseases*	16 (23%)
5	Taylor et al. [28]	*Risk factors for human disease emergence*	16 (23%)
5	Johnson et al. [29]	*Global shifts in mammalian population trends reveal key predictors of virus spillover risk*	16 (23%)

### Convergence of scientific and policy knowledge bases

**Fig. 4:**
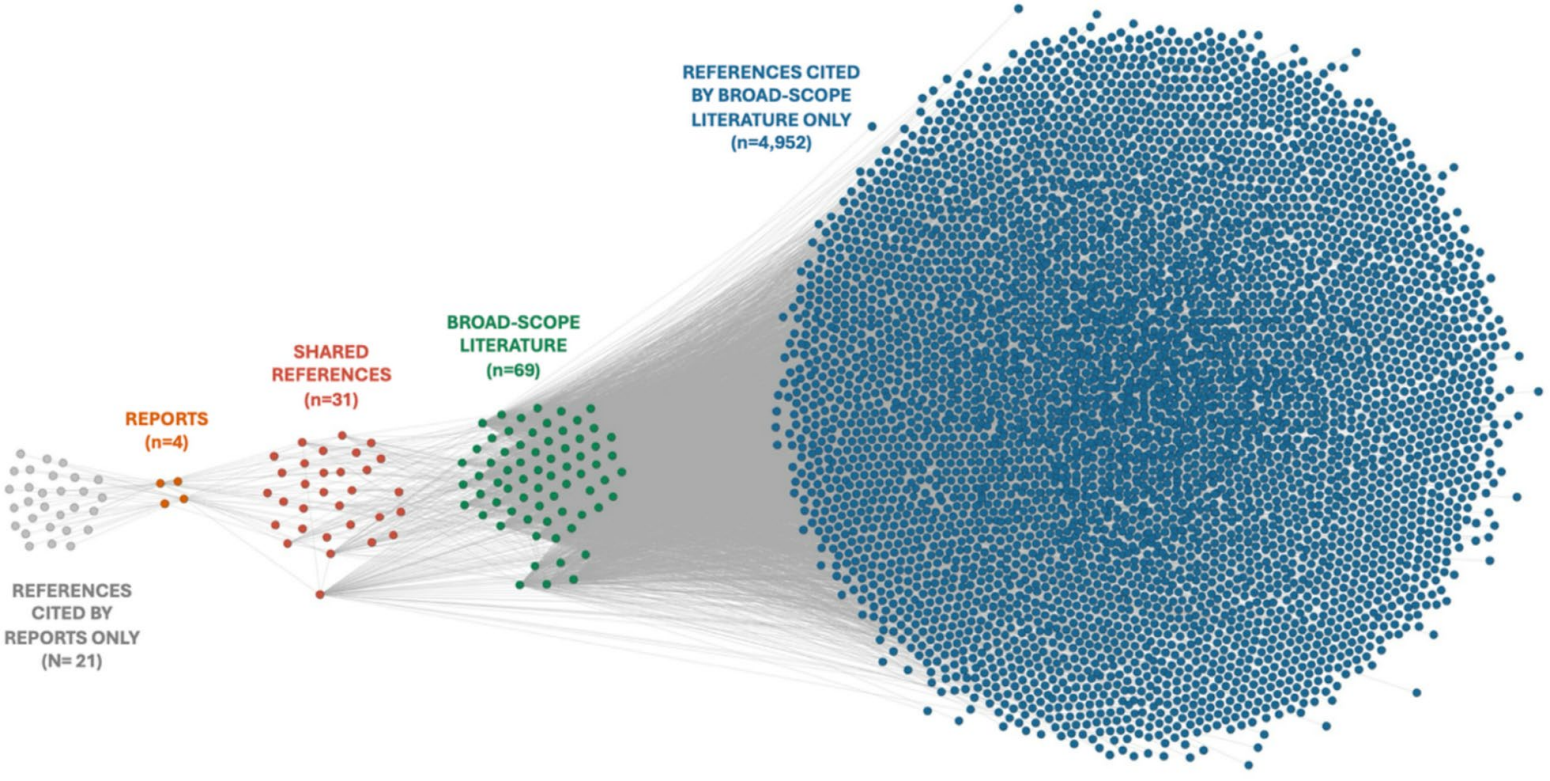
Shared citation network between global policy reports and ZDD broad-scope scientific literature. Nodes indicate scientific publications, reports or cited references. Directed edges indicate citation relationships from either dataset. Nodes were colour-coded according to type of document (grey: references cited only by global reports; orange: global reports; red: references shared by both reports and scientific literature; dark red: broad-scope paper cited by reports; dark green: broad-scope papers; light green: broad-scope papers cited by other broad-scope papers; blue: references unique to the broad-scope scientific literature)

To assess the overlap between scientific literature and global policy, we compared the reference sets from the four global reports and the 69 broad-scope ZDD papers (Fig. 4).

While the two datasets draw on partially distinct bodies of work, key references appear within the shared network, indicating convergence around foundational studies. Of the 59 unique references cited across the global reports, 31 (52%) were also cited by the broad-scope scientific literature, demonstrating substantial convergence in the knowledge base. Among these, two papers – Jones et al. [23] and Taylor et al. [28] – ranked among the five most cited within the scientific dataset, highlighting their central role in both academic and policy-oriented discourses. Only one paper from the broader ZDD dataset – Carlson et al. [30] – was cited exclusively by the reports. The complete shared reference list is presented in Table 4.

**Table 4:** Shared references between global reports and broad-scope ZDD literature

Author	Title
Alirol et al. [31]	*Urbanisation and infectious diseases in a globalised world*
Allen et al. [32]	*Global hotspots and correlates of emerging infectious zoonotic diseases*
Bernstein et al. [1]	*The costs and benefits of primary prevention of zoonotic pandemics*
Carlson et al. [30]	*Climate change increases cross-species viral transmission risk*
Chua et al. [33]	*Anthropogenic deforestation, El Niño and the emergence of Nipah virus in Malaysia*
Dobson et al. [2]	*Ecology and economics for pandemic prevention*
Epstein et al. [34]	*Climate change and emerging infectious diseases*
Grace et al. [35]	*Mapping of poverty and likely zoonoses hotspots*
Himsworth et al. [36]	*Rats, cities, people, and pathogens: a systematic review and narrative synthesis of literature regarding the ecology of rat-associated zoonoses in urban centers*
Hu et al. [37]	*Discovery of a rich gene pool of bat SARS-related coronaviruses provides new insights into the origin of SARS coronavirus*
Hui et al. [38]	*Middle East respiratory syndrome coronavirus: risk factors and determinants of primary, household, and nosocomial transmission*
Jones et al. [23]	*Global trends in emerging infectious diseases*
Kessler et al. [39]	*Changing resource landscapes and spillover of henipaviruses*
Leroy et al. [40]	*Multiple Ebola virus transmission events and rapid decline of Central African wildlife*
Loh et al. [41]	*Targeting transmission pathways for emerging zoonotic disease surveillance and control*
Ma et al. [42]	*The pig as a mixing vessel for influenza viruses: human and veterinary implications*
McFarlane et al. [43]	*Land-use change and emerging infectious disease on an island continent*
McKee et al., [44]	*The ecology of nipah virus in Bangladesh: a nexus of land-use change and opportunistic feeding behavior in bats*
Morand [45]	*Emerging diseases, livestock expansion and biodiversity loss are positively related at global scale*
Morand and Lajaunie [46]	*Outbreaks of vector-borne and zoonotic diseases are associated with changes in forest cover and oil palm expansion at global scale*
Neiderud [47]	*How urbanization affects the epidemiology of emerging infectious diseases*
Olivero et al. [48]	*Recent loss of closed forests is associated with Ebola virus disease outbreaks*
Patz et al. [49]	*Unhealthy landscapes: policy recommendations on land use change and infectious disease emergence*
Patz et al. [50]	*Global climate change and emerging infectious diseases*
Rohr et al. [51]	*Emerging human infectious diseases and the links to global food production*
Shah et al. [52]	*Agricultural land-uses consistently exacerbate infectious disease risks in Southeast Asia*
Smith et al. [53]	*Global rise in human infectious disease outbreaks*
Smolinkski et al. [54]	*Microbial threats to health: emergence, detection and response*
Stephens et al. [55]	*Characteristics of the 100 largest modern zoonotic disease outbreaks*
Taylor et al. [28]	*Risk factors for human disease emergence*
Yuen et al. [56]	*Hendra virus: epidemiology dynamics in relation to climate change, diagnostic tests and control measures*

### Qualitative profile of shared references

**Fig. 5:**
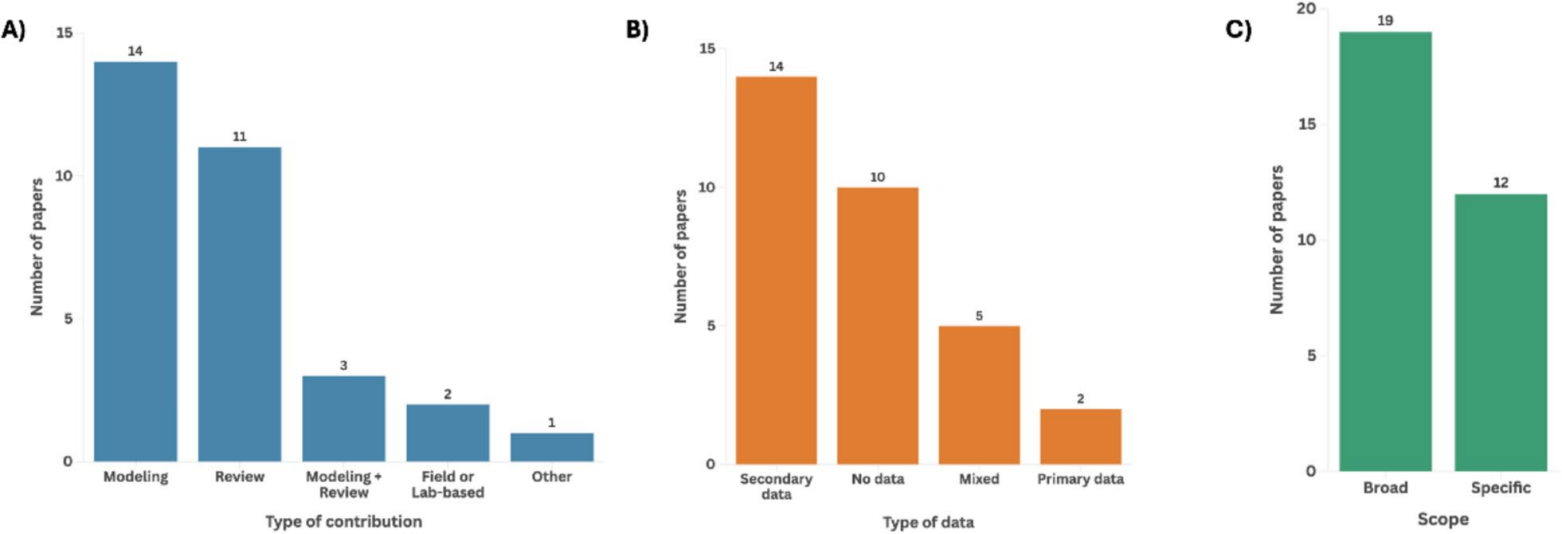
Qualitative profile of the 31 shared references cited by both global policy reports and broad-scope ZDD literature. (**A**) Type of contribution; (**B**) type of data; (**C**) scope

The qualitative analysis of the 31 references cited both by global policy reports and by the broad-scope scientific literature on ZDD revealed that the most common type of intellectual contribution was empirical modelling or simulation studies (45%), followed by review or synthesis papers (35%) and a mix of these two approaches (9%) (Fig. 5A).

Regarding the type of data used, secondary data predominated (45%), while 32% of references did not include empirical data, relying instead on theoretical or argumentative approaches (Fig. 5B). Only 6% were based primarily on original data collection. Most studies using secondary data relied on the Jones et al. [23] database or on the GIDEON database [57].

In terms of thematic scope, 61% of the shared references adopted a broad and integrative perspective on ZDD, exploring general patterns, mechanisms or drivers of zoonotic emergence across systems or regions (Fig. 5C). The remaining 39% focused on specific pathogens, host species or outbreak contexts.

## Discussion

Although ZDD is acknowledged as central to the emergence of infectious diseases, our findings reveal considerable variation in how recent global PPPR reports engage with scientific literature on this topic. Overall, the incorporation of ZDD evidence within the sampled reports appears limited and uneven. Some reports, particularly those by the WB, cite a range of ZDD-related literature, though relying on a partially overlapping set of references. Others include few relevant citations or omit them entirely. This reflects a broader pattern of selectivity in policy documents, which tend to rely on a limited set of sources to support strategic messaging, often at the expense of more recent or interdisciplinary research [58–60]. Such selectivity, though justifiable within the specific functions of policy reports (intended to offer strategic guidance rather than exhaustive literature assessments), runs a risk of narrowing the evidentiary base and limiting the diversity of perspectives informing global strategies.

Citation overlaps between reports also require careful interpretation. In some cases, overlaps may stem from shared contributors or institutional continuity, as seen in the two WB documents [5, 6]. Thus, what may appear as cross-validation or independent convergence could instead reflect the reproduction of the same evidence across documents shaped by similar expert input. This can inflate the apparent weight of specific studies and highlights the importance of distinguishing between consensus that was based on independent validation, and usage, which may result from institutional replication rather than broad epistemic corroboration.

Moreover, the limited number of broad-scope studies highlights the complexity of addressing ZDD as a systemic phenomenon. Most research remains focused on specific pathogens, often constrained by disciplinary or geographic boundaries, making it difficult to identify generalizable patterns. This is understandable given the inherent interdisciplinary of studying ZDD, which requires bridging diverse fields such as virology, ecology, public health and the social sciences [61]. The fragmented structure of the citation network further underscores the challenges of constructing a shared evidence base in a field marked by epistemic diversity and evolving theoretical frameworks. Similar patterns have been observed in other emerging interdisciplinary domains, where conceptual breadth often precedes the formation of integrated research agendas [62, 63] and disciplinary boundaries may hinder synthesis and knowledge translation [64]. These findings suggest that, within the recent literature, research on ZDD is both specialized and conceptually dispersed – rich in the number of empirical studies but limited in its capacity for integration.

An important implication of this lack of integration is that it may obscure or undervalue the complexity inherent in ZDD. While many studies generate important insights into specific mechanisms, their narrow scope can limit the ability to capture cross-cutting drivers, confounders or interactions that could offset or moderate how the findings are interpreted. This suggests a need for additional interdisciplinary, multivariable research capable of connecting different dimensions of ZDD, and for greater awareness among policy-makers of the limitations within their evidence base.

The overlap in citations between global reports and the broad-scope scientific literature suggests a degree of alignment between policy discourse and the academic evidence base. Among the shared references, Jones et al. [23] stand out as keystone publication. By analysing an extensive dataset of outbreaks, the study identified zoonotic pathogens as key drivers of emerging infectious diseases (EID) [23]. Its centrality across both domains likely reflects both its conceptual utility and a so-called Matthew effect: a self-reinforcing cycle where early advantages in visibility or citations lead to disproportionate long-term influence [65]. Previous work has shown that policy uptake of science is often nonlinear and selective, favouring already well-circulated sources [66]. However, this influence is not without caveats: Jones et al. [23] has also been selectively or inaccurately cited in both academic and policy documents [9, 10], including the conflation of detection with incidence, raising questions about how such references are mobilized and interpreted in support of strategic narratives.

Interestingly, nearly half of the references cited in the global reports are absent from the broad-scope literature, and only one of the 69 broad-scope studies was cited by any report. This limited intersection points to a potential disconnect between integrative, emerging research and the sources informing high-level policy. While this may reflect an emphasis on established references, it also highlights an opportunity to strengthen the uptake of emerging, interdisciplinary perspectives in PPPR. In other words, there is a wider range of research available that is not being synthesized into current ZDD policy discourses. Research that could support or challenge current PPPR policy to promote better policy decisions and subsequent outcomes.

Finally, the qualitative profile of the shared references revealed a preference for integrative or predictive forms of evidence, particularly modelling studies, reviews and theoretical contributions based on secondary data. While such approaches offer strategic utility across contexts, they underrepresent context-specific, field-based or critical perspectives. In the case of ZDD, such perspectives may be essential for designing effective and equitable interventions. Moreover, an overreliance on consensus-based or model-driven evidence can obscure key uncertainties, particularly those associated with structural or poorly understood processes [67]. As highlighted in other domains, such as climate policy, explicitly acknowledging uncertainty may be more productive than minimizing it, particularly when developing adaptive strategies in the face of complexity.

### Limitations

This study has several limitations. It focuses on a purposive sample of six influential global reports, which may not reflect the full diversity of policy documents addressing ZDD across institutional or regional contexts. The analysis also relied on Scopus-indexed publications, which may exclude relevant nonindexed literature (including grey literature) that can influence policy. In addition, only English publications were included, thus limiting the range of potential literature for review. The thematic classification of references was conducted manually and involved interpretive judgment, which introduces a degree of subjectivity and potential variability in results. Furthermore, while citation network analysis reveals patterns of citation presence and frequency, it does not capture the interpretive use of scientific evidence or its actual influence on policy formulation.

## Conclusions

This study examined how global PPPR reports cite scientific literature on ZDD, revealing both areas of alignment and significant gaps. While a set of studies – notably Jones et al. [23] – appears to anchor the science–policy interface, the broader landscape of academic research remains only partially reflected in high-level policy discourse. Reports tend to privilege integrative frameworks, modelling approaches and widely circulated sources, while recent, context-specific or interdisciplinary contributions are largely absent. At the same time, the structure of the scientific literature itself – methodologically diverse and conceptually fragmented – poses challenges for integration and synthesis. These structural features limit the potential for building a cohesive knowledge base to inform comprehensive PPPR strategies. Taken together, these findings highlight the value of systematic approaches to scientific evidence used in PPPR policy documents. In complex and uncertain domains such as ZDD, strengthening the science–policy interface requires not only recognizing foundational contributions but also engaging with emerging insights, epistemic diversity and uncertainty. Doing so is essential for building more adaptive, proportionate, equitable and context-sensitive approaches to pandemic preparedness and response.

## Supplementary Information

Supplementary Material 1.

Supplementary Material 2.

## Data Availability

All data generated or analysed during this study are included in this published article and its supplementary information files.

## Abbreviations

EID: Emerging infectious diseases
PPPR: Pandemic prevention, preparedness and response
ZDD: Zoonotic disease dynamics
WB: World Bank
WHO: World Health Organization
G20: Group of Twenty

## Glossary

Broad-scope studies: Multidisciplinary research approaches that investigate cross-cutting factors influencing zoonotic disease spillover or emergence across diverse pathogen–host systems, integrating environmental, societal and health perspectives for pandemic risk reduction.
Citation network analysis: A bibliometric method that maps and analyses citation relationships between documents to identify highly referenced studies, knowledge clusters and patterns of evidence use.
Epistemic diversity: The coexistence of multiple knowledge frameworks, methodological approaches and disciplinary perspectives within a research field, often resulting in varied ways of conceptualizing problems, generating evidence and interpreting findings.
Matthew effect: A cumulative advantage phenomenon where publications with higher initial recognition disproportionately accumulate further citations, resulting in increasing inequality in citation distributions over time.
Pandemic Prevention, Preparedness and Response (PPPR): An integrated framework for reducing infectious disease risks, strengthening health system capacities and managing pandemic threats through surveillance, mitigation strategies and outbreak control measures.
Zoonotic disease dynamics (ZDD): The multidisciplinary study of the processes governing zoonotic pathogen spillover, transmission and emergence, including host–pathogen interactions, infection dynamics at the human–animal interface and epidemiological patterns of disease spread.
Zoonotic spillover: The processes that enable a pathogen from a vertebrate animal to establish infection in a human [3].
